# Concurrent immunoglobulin G4-related disease and hypereosinophilia with persistent fever and simultaneous acute multi-organ involvement

**DOI:** 10.1093/rap/rkad078

**Published:** 2023-09-22

**Authors:** Takashi Nawata, Kohei Goda, Motoaki Tsutsui, Tadayoshi Ikegami, Hideaki Akase, Shinichi Okuda, Fumiaki Nakao, Takeshi Ueyama, Noriko Uesugi, Yasuhiro Ikeda

**Affiliations:** Department of Medicine and Clinical Science, Yamaguchi University Graduate School of Medicine, Ube, Japan; Department of Rheumatology, Yamaguchi Prefectural Grand Medical Center, Hofu, Japan; Department of Cardiology, Yamaguchi Prefectural Grand Medical Center, Hofu, Japan; Department of Cardiology, Yamaguchi Prefectural Grand Medical Center, Hofu, Japan; Department of Nephrology, Yamaguchi Prefectural Grand Medical Center, Hofu, Japan; Department of Cardiology, Yamaguchi Prefectural Grand Medical Center, Hofu, Japan; Department of Cardiology, Yamaguchi Prefectural Grand Medical Center, Hofu, Japan; Department of Cardiology, Yamaguchi Prefectural Grand Medical Center, Hofu, Japan; Department of Cardiology, Yamaguchi Prefectural Grand Medical Center, Hofu, Japan; Department of Pathology, Faculty of Medicine, Fukuoka University, Fukuoka, Japan; Department of Cardiology, Yamaguchi Prefectural Grand Medical Center, Hofu, Japan

Key message

- Severe hypereosinophilia may be a modifying factor in IgG4-related disease.

Dear Editor, IgG4-related disease (IgG4-RD) is a fibroinflammatory disease characterized by elevated serum IgG4 levels and organ enlargement [1]. Following the initial report of IgG4-RD, the disease has been recognized by clinicians worldwide [2]. However, the pathological mechanisms underlying IgG4-RD remain unclear, and IgG4-RD-mimicking diseases can be problematic in clinical practice [3]. Herein, we report a case of IgG4-RD accompanied by various atypical findings that appeared to be caused by over-activation of an allergic reaction.

A 57-year-old Japanese man was admitted to the Yamaguchi Prefectural Grand Medical Center with persistent fever of ∼38°C (sometimes, >38°C), which had developed 2 months before admission. Laboratory examinations at the previous hospital revealed a significantly elevated serum IgG4 level (1510 mg/dl). Based on these findings, IgG4-RD was suspected, and the patient was referred to our hospital.

Upon admission, the patient presented with jaundice of the skin and conjunctiva. His body temperature was 37.3°C. The patient’s complete blood cell count revealed eosinophilia (maximum count during his clinical course: 2997/µl), while laboratory examinations revealed an elevated serum CRP level (1.99 mg/dl). Liver and renal involvement were also observed (serum levels of aspartate transaminase, 51 U/l; alanine transaminase, 45 U/l; alkaline phosphatase, 453 U/l; γ-glutamyltransferase, 400 U/l; total bilirubin, 2.1 mg/dl; and creatinine, 1.75 mg/dl). Serum levels of amylase and lipase were within normal limits (51 U/l and 33 U/l, respectively), whereas those of IgG, IgG4 and IgE were elevated [4897 mg/dl, 2430 mg/dl and 3440 IU/ml (normal range: within 360.9 IU/ml), respectively]. The patient tested negative for ANA, MPO-ANCA, and PR3-ANCA. Serum levels of C3 and C4 were significantly decreased (62 mg/dl and 2 mg/dl, respectively). The patient’s bone marrow biopsy results did not indicate haematologic disease, and chromosomal abnormality was not found. ^18^F-Fluorodeoxyglucose (^18^F-FDG) PET-CT revealed ^18^F-FDG accumulation in the lacrimal glands, salivary glands, lymph nodes, pancreas, prostate and bilateral kidneys (Fig. 1A). Furthermore, CT did not show pancreatic enlargement. Biopsy of the right submandibular gland revealed infiltration of plasma cells, which were almost universally IgG4 positive (Fig. 1B). In addition, biopsy of the right kidney revealed infiltration of IgG4-positive plasma cells, accompanied by storiform fibrosis and obliterative phlebitis (Fig. 1C, D). Based on these findings, the patient was diagnosed with IgG4-RD. Infiltration of eosinophils into the interstitium of the kidney was also observed (Fig. 1E). In addition, the patient experienced anaphylactic shock accompanied by decreased blood pressure (107/90 mmHg), tachypnoea (24 breaths/min), dyspnoea, wheeze on chest auscultation, and cold sweats after meals during the period of scrutiny, despite having no allergic history.

**Figure 1.**
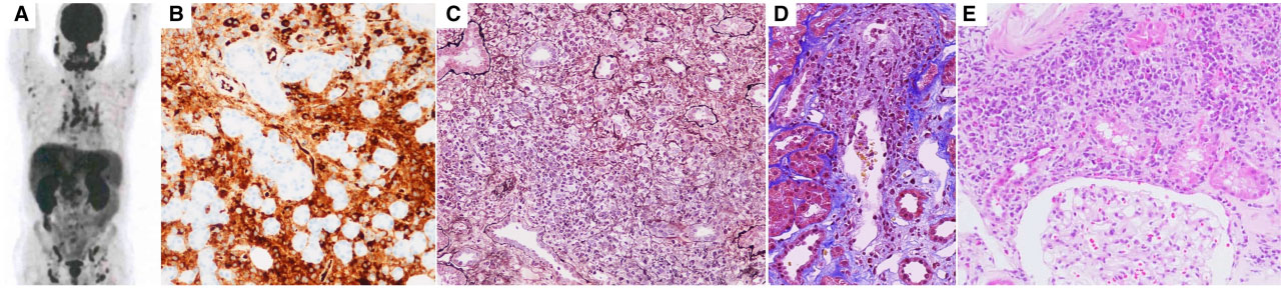
Imaging and histological findings of the patient. (**A**) ^18^F-Fluorodeoxyglucose (^18^F-FDG) PET-CT showing accumulation of ^18^F-FDG in the lacrimal glands, salivary glands, lymph nodes, pancreas, prostate and bilateral kidneys. (**B**) Biopsy of the right submandibular grand showing infiltration of IgG4-positive plasma cells. (**C**) Biopsy of the right kidney showing storiform fibrosis (Periodic Acid Methenamine Silver staining). (**D**) Biopsy of the right kidney showing obliterative phlebitis (Elastica Masson staining). (**E**) Biopsy of the right kidney showing infiltration of eosinophils in the interstitium of the kidney (Haematoxylin and Eosin staining)

We concluded that the patient’s symptoms were attributable to the overlap of IgG4-RD and hypereosinophilia. He was treated with a 3 day course of CS pulse therapy (methylprednisolone, 1 g daily), followed by oral prednisolone (initial dose, 50 mg daily), after which his fever disappeared. In addition, improvements were noted in fever, eosinophilia, liver involvement and renal involvement.

This patient showed severe eosinophilia (almost 3000/µl), persistent fever and acute multi-organ involvement. Previous research has indicated that patients with IgG4-RD often show a mild to moderate degree of eosinophilia [4]. However, the 2019 ACR/EULAR classification criteria for IgG4-RD defines severe peripheral eosinophilia (>3000/µl) and fever as exclusion criteria for IgG4-RD [5]. Therefore, other differential diagnoses should be considered when clinicians encounter patients with high serum IgG4 levels, severe eosinophilia and fever [5]. Furthermore, IgG4-RD often causes chronic fibrotic inflammation [6]. Therefore, simultaneous acute multi-organ involvement is an atypical finding of IgG4-RD.

The patient’s pathological and PET/CT findings suggested IgG4-RD. However, considering his atypical findings of IgG4-RD, the pathology of our patient seemed to indicate the presence of composite factors in addition to IgG4-RD. Although our patient had no history of allergies before hospital admission, he experienced anaphylactic shock immediately before treatment with CSs. This event and the high elevation in serum eosinophil count suggest an overlap of IgG4-RD with hypereosinophilia. Thus, we hypothesized that over-activation of allergic reactions by unexpected causes can occur in patients with a chronic course of IgG4-RD, which might consequently lead to severe eosinophilia and simultaneous acute multi-organ involvement accompanied by fever.

The present case suggests that clinicians should consider the existence of modifying factors, such as hypereosinophilia, when patients present with atypical IgG4-RD, even if pathological and imaging findings are consistent with those of IgG4-RD. Few cases of IgG4-RD associated with severe hypereosinophilia and/or eosinophilic ascites have been reported [4, 7, 8]. The pathological findings of the renal biopsy suggest that the renal involvement in our patient was caused by eosinophilic interstitial nephritis. We also believe that the liver involvement was caused by hypereosinophilia, similar to that observed in a previous report [8]. However, the pathological and clinical significance of severe hypereosinophilia in patients with IgG4-RD remains unclear. Further studies are needed to reveal the pathology of atypical IgG4-RD associated with severe hypereosinophilia.

## Data Availability

The authors declare that all the relevant data supporting the findings of this case report are available within the article.
